# Mitophagy and Neuroinflammation: A Compelling Interplay

**DOI:** 10.2174/1570159X20666220628153632

**Published:** 2023-05-18

**Authors:** Nikolaos Charmpilas, Evandro Fei Fang, Konstantinos Palikaras

**Affiliations:** 1 Institute for Genetics and Cologne Excellence Cluster on Cellular Stress Responses in Ageing-Associated Diseases (CECAD), University of Cologne, Cologne, Germany;; 2 Department of Clinical Molecular Biology, University of Oslo, Oslo, Norway;; 3 Akershus University Hospital, Lørenskog, Norway;; 4 The Norwegian Centre on Healthy Ageing (NO-Age), Oslo, Norway;; 5 Department of Physiology, Medical School, National and Kapodistrian University of Athens, Athens, Greece

**Keywords:** Ageing, energy homeostasis, immunity, inflammation, metabolism, mitochondria, mitophagy, neurodegeneration

## Abstract

Mitochondria are the main sites of energy production and a major source of metabolic stress. Not surprisingly, impairment of mitochondrial homeostasis is strongly associated with the development and progression of a broad spectrum of human pathologies, including neurodegenerative disorders. Mitophagy mediates the selective degradation of damaged organelles, thus promoting cellular viability and tissue integrity. Defective mitophagy triggers cellular senescence and prolonged neuroinflammation, leading eventually to cell death and brain homeostasis collapse. Here, we survey the intricate interplay between mitophagy and neuroinflammation, highlighting that mitophagy can be a focal point for therapeutic interventions to tackle neurodegeneration.

## 1 INTRODUCTION

The degradation of malfunctioning and superfluous mitochondria *via* macroautophagy hereafter referred to as mitophagy, is a core cellular quality control process, which safeguards the functionality of the mitochondrial network and allows its adaptation to the ever-changing physiological demands [1, 2]. In the best-studied mitophagy paradigm, membrane potential dissipation impedes the import of PTEN-induced kinase 1 (PINK1) into mitochondria, favoring its stabilization to the outer mitochondrial membrane (OMM) [3]. Subsequently, PINK1 phosphorylates ubiquitin moieties on several protein substrates, as well as the E3 ubiquitin-protein ligase Parkin, which can then be recruited and activated in the OMM’s vicinity [4]. Parkin in turn ubiquitinates numerous OMM proteins, allowing their recognition by bona fide autophagic adaptors en route to degradation in the lysosomes [5]. In coordination with PINK1/Parkin ubiquitin-dependent mitophagy, distinct receptors facilitate the elimination of mitochondria *via* direct interaction of their LC3-interacting region (LIR) motifs with the LC3 and GABARAB proteins that are anchored to the autophagosomal membranes. Prominently, NIP3-like protein X(NIX) fine-tunes mitophagy during development, while BCL2 interacting protein 3 (BNIP3) and FUN14 domain-containing 1 (FUNDC1) mediate mitochondrial turnover in response to stressful insults [6-9]. Interestingly, mitophagy receptors are not restricted to the OMM, since Prohibitin 2 (PHB2), a protein localized in the inner mitochondria membrane (IMM), and cardiolipin can be exposed to the cytoplasm, interact with LC3 and act as mitophagy receptors when the OMM integrity is perturbed [10-12]. Overall, mitophagy serves as an integral surveillance mechanism that preserves mitochondrial homeostasis under physiological conditions and upon stress.

Mitophagy defects have been associated with a plethora of diseases, especially affecting cells, tissues, and organs with high energetic demands. As such, neurons are highly dependent on mitochondria to perform their specialized functions, such as axonal transport and neurotransmitter secretion [13]. However, as revealed by the use of elegant mitophagy reporters, distinct neuronal sub-populations and regions within the mammalian brain display prominent differences *in vivo*, with the dentate gyrus and the Purkinje cell layer of the cerebellum having especially elevated baseline mitophagy levels [14]. It is noteworthy that prevalent neurodegenerative pathologies in humans, such as Alzheimer’s disease (AD), Parkinson’s disease (PD), and amyotrophic lateral sclerosis (ALS) are accompanied by aberrant mitophagy and/or impaired mitophagic flux [15]. Post-mortem AD human hippocampal samples exhibit abnormal mitochondrial cristae morphology compared to their wild-type counterparts, while induced pluripotent stem cell (iPSCs)-derived AD neurons display impaired mitochondrial morphology and defects in autophagy initiation [16]. Strikingly, supplementation with chemical inducers of mitophagy is sufficient to mitigate cognitive deficits in mouse and nematode models of AD [16, 17]. These findings suggest that dysfunctional mitophagy may underlie the manifestation and progression of AD and that its induction may constitute a promising therapeutic strategy for AD confrontation. On the other hand, there are also reported cases where uncontrolled mitophagy proves detrimental and aggravates neuronal cell death [18-20]. For instance, a recent report documented that the administration of antipsychotic drugs can ameliorate pathological hallmarks of multiple sclerosis (MS) partially *via* limiting mitophagy [21]. Moreover, uncontrolled mitophagy reduces the mitochondrial population in the axons of the retinal ganglion cells (RGCs) triggering their cell death in a mouse model of mitochondrial optic neuropathy [19, 20]. Collectively, these studies demonstrate that mitophagy should be strongly controlled to preserve neuronal function along with ageing, since its impairment or deregulation can undermine brain homeostasis, thus promoting neuropathology and neurodegeneration.

Neuroinflammation denotes the inflammatory responses within the central nervous system (CNS, *i.e*., the brain, the retina, and the spinal cord) following traumatic brain or spinal cord injury, infection, ischemia, diabetes, and intraocular pressure or as a corollary of autoimmunity and ageing itself. Neuroinflammation is mainly triggered by the release of pro-inflammatory cytokines, chemokines, second messengers (*e.g*., nitric oxide and prostaglandins), and reactive oxygen species (ROS). The glial cells, such as microglia and astrocytes, are CNS-resident gatekeepers of immunity and are considered as central modulators of neuroinflammation; nevertheless, when the integrity of the blood-brain barrier is compromised, endothelial cells and peripheral monocytes also contribute to its progression [22]. Although neuroinflammation can occasionally exert neuroprotective effects, promoting repair from acute CNS injury, when prolonged it can be maladaptive and exacerbate human pathology. This concept becomes particularly evident in AD patients, where persistent microglial activation drives neuronal dysfunction and death, thereby aggravating cognitive decline at the late stages of AD [23].

**Fig. (1):**
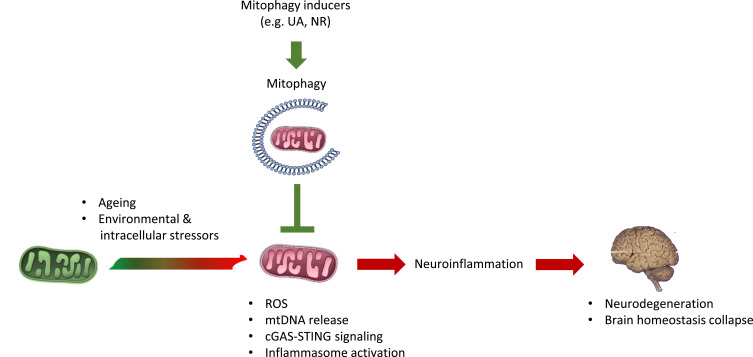
Mitophagy enhancement sustains mitochondrial function and confers neuroprotection. Fine-tuned mitochondrial activity is a prerequisite for the maintenance of cellular and tissue homeostasis. Damaged mitochondria lead to elevated ROS production, mtDNA release, cGAS-STING signaling, inflammasome activation, and subsequently neuroinflammation. Mitophagy-inducing agents, such as urolithin A (UA) and nicotinamide riboside (NR), restore mitochondrial function, inhibit prolonged inflammation, and protect against neurodegeneration.

Apart from being a crucial site for mitochondria recognition by autophagosomes en route to their elimination, the OMM can also act as a signaling platform for the activation of inflammatory responses [24]. Notably, the mitochondrial antiviral-signaling adaptor protein (MAVS), which is activated upon sensing invading double-stranded viral RNAs by retinoic acid-inducible gene I (RIG1) and lies upstream of NF-κB and the interferon regulatory transcription factors (IRFs), is activated in the proximity of the OMM by mitochondrial ROS [25]. A recent study demonstrated that endogenous insults, such as double-strand breaks in the mitochondrial DNA (mtDNA), can also stimulate a MAVS-dependent immune response, mediated by the release of mitochondrial RNAs in the cytosol [26]. However, the inflammatory potential of NF-κB is thought to be restricted by p62/SQSTM1-mediated mitophagy, which eliminates damaged mitochondria in macrophages. In this context, Parkin is required for p62/SQST-1 recruitment and the subsequent elimination of mitochondria, thereby limiting signals that promote the release of the pro-inflammatory cytokine interleukin-1 (IL-1) from macrophages [27]. The link between mitophagy and neuroinflammation remained largely ambiguous since several studies using mouse models deficient for either PINK1 or Parkin failed to recapitulate phenotypes reminiscent of PD pathology [28-31]. Moreover, the serum cytokine levels in those mice remained relatively unaltered, when compared to their wild-type counterparts. Yet, in another study, elevated levels of pro-inflammatory cytokines, such as tumor necrosis factor -α (TNF-α), interleukin -6, and -1β(IL-6 and IL-1β), were detected in accurately prepared cortical slices from PINK1 knockout mice [32]. In contrast to rodent mutants for PINK1 and Parkin, human blood macrophages isolated from PD patients with *PARKIN* mutations exhibit an overactivation of the NLR family pyrin domain containing 3 (NLRP3) inflammasome upon administration of potent inflammatory stimulators, such as lipopolysaccharide-nigericin or lipopolysaccharide-ATP [33]. Moreover, humans carrying the *PINK1G309D* mutation, associated with early-onset familial PD, display elevated expression of vascular cell adhesion molecule 1 (VCAM-1), which aids the attachment of human monocytes to brain endothelial cells [34]. Another study reported that mutations in *PARKIN* augmented the release of the pro-inflammatory cytokines (IL-6 and IL-1β) and the chemokines CCL2 and CCL4 in the serum of humans suffering from idiopathic PD. Of particular note, elevated IL-6 levels were also detected in individuals with monoallelic *PARKIN* mutations, even though they are fully asymptomatic, demonstrating that the acquisition of a pro-inflammatory profile is not merely an outcome, but rather precedes disease manifestation [31]. These findings are in congruence with the notion that PINK1-mediated mitophagy dampens the release of pro-inflammatory cytokines from microglia in the pathological context of AD. Vice versa, supplementation of urolithin A or nicotinamide riboside (NR), potent mitophagy inducers, is sufficient to reduce the total levels of the NLRP3 inflammasome, apoptosis, and cellular senescence, as well as the pro-inflammatory cytokines TNF-α and IL-6 in an AD mouse models [16, 35]. The uncontrolled induction of cyclic GMP-AMP synthase (cGAS) and stimulator of interferon genes (STING) are strongly associated with DNA damage, senescence, and neurodegeneration [36]. cGAS-STING signaling is found to be elevated in both AD and ataxia telangiectasia (A-T) mouse models underscoring its detrimental role in neuroinflammation and neurodegeneration [35, 37]. Interestingly, NR treatment restored the levels of cGAS-STING signaling, thereby suppressing senescence, aberrant inflammatory response, and motor and cognitive functions in both AD and A-T mouse models [35, 37]. Collectively, the aforementioned studies indicate that robust mitophagy limits neuroinflammation with critical implications for neuronal homeostasis (Fig. **1**).

Accumulating evidence implicates the PINK1/Parkin mitophagy axis in inflammatory signaling with particular relevance to neuronal homeostasis and function. We anticipate that future studies will shed light on novel nodes on the interaction between mitophagy receptors that fulfill distinct cellular requirements and inflammatory modulators. During the last decade, several versatile imaging methods have been generated to assess mitochondrial turnover in various cell types and organisms *in vivo* [14, 38-41]. These mitophagy reporters should be combined with disease models and be used as screening platforms to uncover novel mitophagy modulators that could confer neuroprotection and animal survival. In light of the ever-increasing life expectancy of the human population, these studies can foster the development of efficient therapeutic approaches to mitigate excessive neuroinflammation as a means to decelerate the progression of neurodegenerative diseases affecting the elderly.

## LIST OF ABBREVIATIONS

AD: Alzheimer’s Disease
ALS: Amyotrophic Lateral Sclerosis
CNS: Central Nervous System
IMM: Inner Mitochondria Membrane
iPSCs: Induced Pluripotent Stem Cell
MS: Multiple Sclerosis
mtDNA: Mitochondrial DNA
NR: Nicotinamide Riboside
OMM: Outer Mitochondrial Membrane
PD: Parkinson’s Disease
RGCs: Retinal Ganglion Cells
ROS: Reactive Oxygen Species
